# Identification of a Novel TGF-β-Binding Site in the Zona Pellucida C-terminal (ZP-C) Domain of TGF-β-Receptor-3 (TGFR-3)

**DOI:** 10.1371/journal.pone.0067214

**Published:** 2013-06-27

**Authors:** Uschi Diestel, Marcus Resch, Kathrin Meinhardt, Sigrid Weiler, Tina V. Hellmann, Thomas D. Mueller, Joachim Nickel, Jutta Eichler, Yves A. Muller

**Affiliations:** 1 Lehrstuhl fuer Biotechnik, Department of Biology, Friedrich-Alexander-University Erlangen-Nuremberg, Erlangen, Germany; 2 Julius-von-Sachs-Institut fuer Biowissenschaften, Lehrstuhl fuer Botanik I, University of Wuerzburg, Wuerzburg, Germany; 3 Lehrstuhl fuer Tissue Engineering und Regenerative Medizin, University of Wuerzburg, Wuerzburg, Germany; 4 Department of Chemistry and Pharmacy, Friedrich-Alexander-University Erlangen-Nuremberg, Erlangen, Germany; Griffith University, Australia

## Abstract

The zona pellucida (ZP) domain is present in extracellular proteins such as the zona pellucida proteins and tectorins and participates in the formation of polymeric protein networks. However, the ZP domain also occurs in the cytokine signaling co-receptor transforming growth factor β (TGF-β) receptor type 3 (TGFR-3, also known as betaglycan) where it contributes to cytokine ligand recognition. Currently it is unclear how the ZP domain architecture enables this dual functionality. Here, we identify a novel major TGF-β-binding site in the FG loop of the C-terminal subdomain of the murine TGFR-3 ZP domain (ZP-C) using protein crystallography, limited proteolysis experiments, surface plasmon resonance measurements and synthetic peptides. In the murine 2.7 Å crystal structure that we are presenting here, the FG-loop is disordered, however, well-ordered in a recently reported homologous rat ZP-C structure. Surprisingly, the adjacent external hydrophobic patch (EHP) segment is registered differently in the rat and murine structures suggesting that this segment only loosely associates with the remaining ZP-C fold. Such a flexible and temporarily-modulated association of the EHP segment with the ZP domain has been proposed to control the polymerization of ZP domain-containing proteins. Our findings suggest that this flexibility also extends to the ZP domain of TGFR-3 and might facilitate co-receptor ligand interaction and presentation *via* the adjacent FG-loop. This hints that a similar C-terminal region of the ZP domain architecture possibly regulates both the polymerization of extracellular matrix proteins and cytokine ligand recognition of TGFR-3.

## Introduction

Transforming growth factor-β receptor type 3 (TGFR-3), also known as betaglycan, is an ubiquitously expressed cell surface proteoglycan that serves as a co-receptor for members of the TGF-β family of cystein knot growth factors, *i.e.* TGF-βs, activins, inhibins, growth differentiation factors (GDFs) and the bone morphogenetic proteins (BMPs). TGFR-3 promotes important signaling events like growth regulation, migration, apoptosis, and differentiation [1], [2]. Many TGF-β family members bind to TGFR-3 first, and this interaction subsequently facilitates the formation of a ternary signaling complex between the growth factor and the receptors TGFR-1 and TGFR-2. However, the molecular mechanism by which the co-receptor TGFR-3 initiates and facilitates the formation of the signaling competent complex is currently not well understood [1], [2].

**Figure 1:**
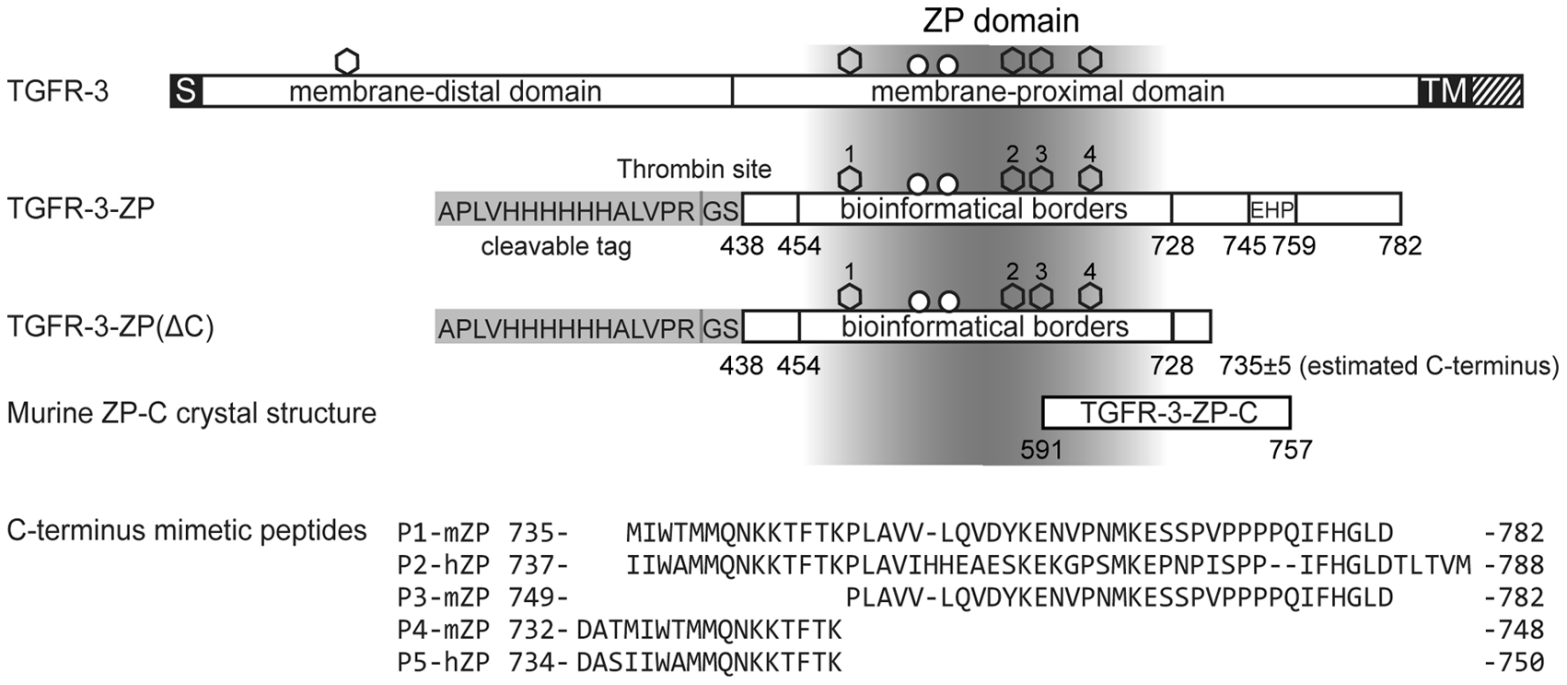
Domain organization and features of murine TGFR-3 and variants. First line: Domain representation of wild-type full-length TGFR-3 emphasizing the membrane-distal and –proximal domain in the TGFR-3 ectodomain. The transmembrane-domain (TM), the signal sequence (S), GAG chains (circles) and potential N-glycosylation sites (hexagons) are also indicated. Second line: The recombinant variant TGFR-3-ZP used in this study comprises residues 438–782 of murine TGFR-3, includes the EHP and features a thrombin-cleavable N-terminal His-tag. The bioinformatically delineated ZP core domain (residues 454–728) is highlighted by a shaded area [9]. In variant TGFR-3-ZP(234) (not shown) the asparagine residues from three out of four potential N-glycosylation sites were mutated to glutamines. Third line: In variant TGFR-3-ZP(ΔC) C-terminal residues were cleaved-off by limited proteolysis. Fourth line: ZP-C fragment as observed in the crystal structure. Subsequent lines display the sequences of the C-terminus mimetic peptides P1 to P5 of human and murine receptor mimetic peptides of TGFR-3-ZP investigated in this study.

The 785 residue-long ectodomain of TGFR-3 can be subdivided into two halves of similar lengths (Fig. 1). Mutagenesis studies showed that both the membrane-distal N-terminal half and the membrane-proximal C-terminal half contribute to growth factor binding [3]–[7]. The C-terminal half contains next to a bioinformatically delineated ZP core domain (residues 454 to 728, Fig. 1) a so-called external hydrophobic patch (EHP) that is part of a stretch of amino acids that connects the ZP domain to the membrane-spanning segment in TGFR-3 [8]. ZP domains were described first for the eponymous zona pellucida proteins ZP1, ZP2 and ZP3 and were later also identified in TGFR-3 and the related protein endoglin [9], [10]. In recent years, structural investigations provided insight into the ZP domain architecture and revealed that the ZP domain itself can be divided into two further subdomains of similar topology, called ZP-N and ZP-C [8]. So far, crystals structures of both the full-length and the N-terminal ZP subdomain (ZP-N) have been determined for ZP3 as well as the C-terminal subdomain (ZP-C) of TGFR-3 [11]–[13].

It is currently assumed that the EHP segment has important regulatory functions and controls the polymerization of ZP domain-containing proteins such as the zona pellucida proteins, tectorins and uromodulin [8]–[11], [13]–[15]. In all ZP structures solved to date, the EHP segment folds back onto the ZP-C subdomain *via* a flexible FG-loop [11], [13]. As a result, the EHP segment is incorporated as a β-strand G into the immunoglobulin-like β-sandwich fold of ZP-C. Polymerization was proposed to be controlled either by a *trans*-association of the EHP segment and/or from the proteolytic removal of the EHP segment [8], [11], [13]. Additional structural and functional data would be highly beneficial to better understand ZP domain function and in particular how this domain is able to fulfil diverse functional roles, spanning from polymerization to growth-factor presentation.

Here, we present a crystal structure analysis of the murine TGFR-3 ZP-C subdomain, limited proteolysis experiments, surface plasmon resonance measurements and studies with receptor mimetic peptides that allowed us to identify a novel major TGF-β-binding site in TGFR-3. This site maps onto the FG-loop of the ZP-C subdomain. When comparing the murine ZP-C structure with the recently determined rat ZP-C structure, we observe a 4-residue shift in β-strand G registration [13]. Since β-strand G contains the EHP segment and immediately follows the FG-loop, these studies highlight that regions that have previously been recognized as important for the controlled polymerization of ZP domain-containing proteins represent also important determinants for growth factor recognition in TGFR-3.

## Materials and Methods

### Cloning of Murine TGFR-3-ZP

In the murine TGFR-3-ZP construct used throughout this work, the bioinformatically predicted ZP domain of TGFR-3 (residues 438 to 728) was extended by 54 residues at the C-terminus (729 to 782) (Fig. 1) [9]. We refer to the first segment as the ZP core domain, whereas the extended C-terminus also incorporates the recently characterized EHP [8], [11], [13]. TGFR-3-ZP was cloned into a modified pCEP-Pu/BM40 vector for protein production in HEK293 EBNA cells (Invitrogen, Karlsruhe, Germany) [16]. The amino-acid numbering in this work adheres to UniProtKB/TrEMBL accession number O88393. It includes the N-terminal signal peptide. In the TGFR-3-ZP construct a mouse BM40 signal sequence enables secretion of the protein into the medium and is followed by a thrombin-cleavable hexa-histidin tag. The cDNA of murine TGFR-3-ZP (gift of Prof. Guy Richardson, University of Sussex) was amplified using the forward primer 5′-CTAGCTAGCCCTGGTGCCGCGCGGCAGCAGAGAGCCAGAAGAAGTG-3′ and the reverse primer 5′-GTTTTTCTCGAGTCACTAGTCCAGGCCGTGGAAAAT-3′. The PCR products were purified and inserted into the multiple cloning site of the vector using *Nhe*I and *Xho*I restriction endonucleases (New England BioLabs, Schwalbach, Germany).

### Mutagenesis of TGFR-3-ZP

In order to obtain a reduced and homogeneous level of *N*-glycosylation in the protein, three of four potential *N*-glycosylation sites within TGFR-3-ZP (N570 (position 2), N589 (position 3), N696 (position 4)) were mutated to glutamines yielding variant TGFR-3-ZP(234). Mutation of N491 (position 1) did not allow for stable expression of the protein. Site directed mutagenesis was achieved performing several rounds of a two stage PCR protocol [17]. Primer pairs are ‘N(570)Q_forw’ 5′-GCTGGAGTGGTAGTGTTTCAGTGCAGCTTGCGGCAGC-3′ and ‘N(570)Q_rev’ 5′-GCTGCCGCAAGCTGCACTGAAACACTACCACTCCAGC-3′; ‘N(589)Q_forw’ 5′-GGACCAGCTCGATGGACAGGCTACCTTCAATATGGAGC-3′ and ‘N(589)Q_rev’ 5′-GCTCCATATTGAAGGTAGCCTGTCCATCGAGCTGGTCC-3′; ‘N(696)Q_forw’ 5′-GTGTTCAAGTCCTGTTCCAGACCTCCCTGCTCTTCCTGC-3′ and ‘N(696)Q_rev’ 5′-GCAGGAAGAGCAGGGAGGTCTGGAACAGGACTTGAACAC-3′. The underlined bases indicate the N to Q codon exchange. Mutations were confirmed by DNA sequencing.

### Recombinant Production and Purification of TGFR-3-ZP Variants

Human embryonic kidney cells constitutively expressing the nuclear antigen-1 protein from the Epstein-Barr virus (HEK293 EBNA cells) were cultivated at 37°C and 5% (*v*/*v*) CO_2_ in Dulbecco’s modified Eagle’s medium Hams F12 supplemented with 10% (*v*/*v*) newborn calf serum, 584.6 µg • ml^-1^ L-glutamine (non-animal source, Sigma-Aldrich, Schnelldorf, Germany), 500 µg • ml^-1^ G-418 sulfate (Calbiochem, Schwalbach, Germany), 100 U • ml^-1^ penicillin and 100 µg • ml^-1^ streptomycin (PenStrep stock solution from Gibco, Karlsruhe, Germany). Cells transfected with Lipofectamine 2000 (Invitrogen) and the vector containing the gene encoding either for TGFR-3-ZP or TGFR-3-ZP(234) were selected with 4 µg • ml^-1^ puromycin (Calbiochem) for stable clones [18]. Supernatants containing the secreted target proteins were collected after replacing growth medium by serum free medium. TGFR-3-ZP and TGFR-3-ZP(234) were purified using a Ni-Sepharose High Performance column (GE Healthcare, Freiburg, Germany) with a binding/washing buffer A containing 20 mM sodium phosphate, pH 7.4, 15 mM imidazole and 0.5 M sodium chloride. Elution buffer B contained in addition 0.5 M imidazole. Proteolytic cleavage with 5 NIH units of thrombin (Sigma-Aldrich) per mg protein fully removed the N-terminal hexa-histidin tag within 16 hours at 20°C. As a final purification step, a size exclusion chromatography (SEC) step was applied using a Superdex 200 16/60 size exclusion column (GE Healthcare) with a buffer containing 20 mM HEPES, pH 7.5, and 150 mM NaCl. Protein concentrations were determined spectrophotometrically at 280 nm using a theoretically-derived molar extinction coefficient of 26,500 M^-1^ cm^–1^ and a molecular weight of 39.6 kDa for both TGFR-3-ZP and TGFR-3-ZP(234). The hexa-histidine tagged proteins were detected by immunoblotting with an anti-penta-His primary antibody (QIAGEN, Hilden, Germany).

### Limited Proteolysis of TGFR-3-ZP and TGFR-3-ZP(234)

In order to gain insight into the TGFR-3-ZP domain stability, we performed limited proteolysis experiments with the unspecific serine protease proteinase K (Sigma-Aldrich) [19]. The protein solutions were adjusted to a concentration of 1 mg • ml^-1^ in gel filtration buffer and incubated with 3 µg proteinase K per mg protein at 20°C. After 4 to 5 hours, 5 mM AEBSF Hydrochloride (AppliChem, Darmstadt, Germany) were added to stop proteolysis. For analysis, the samples were boiled in SDS sample buffer and examined by SDS gel electrophoresis [20]. In order to isolate larger amounts of the proteolytically truncated variants termed TGFR-3-ZP(ΔC) and TGFR-3-ZP(234,ΔC) from hereon, the cleavage step was followed by an additional size exclusion chromatography step using the same buffers and conditions as described above.

Limited proteolysis experiments were also performed at an analytical level in the presence of human TGF-β2 (gift of Prof. Walter Sebald, Department of Physiological Chemistry II, University of Wuerzburg) under the assumption that murine and human TGF-β2 bind with similar affinity to murine TGFR-3-ZP (see below). Equimolar ratios of receptor and ligand were used, and the concentrations adjusted as described above for the single proteins.

### Circular Dichroism and Dynamic Light Scattering Measurements

Circular dichroism (CD) measurements were performed at 20°C in 50 mM potassium phosphate buffer (pH 7.5) at a protein concentration of 1.2 µM with a Jasco J-810 spectropolarimeter (Jasco, Tokyo, Japan) and a cuvette with 0.1 cm path length. Spectra were recorded from 185 to 260 nm, corrected for the phosphate buffer and accumulated five times with a band width of 2.0 nm. The sensitivity was 100 mdeg, the scan speed 20 nm • min^-1^, the time response 1 sec and the data pitch 0.1 nm.

For dynamic light scattering TGFR-3-ZP (2 mg • ml^-1^, 20 mM HEPES, pH 7.4) was passed through a 0.22 µm filter and centrifuged at 10,000 g for 30 min before 20 µl were loaded into a quartz microcuvette. Ten measurements were accumulated at 20°C using a DynaPro Titan instrument (Wyatt Technology Corporation, Santa Barbara, California, USA). Measurements were repeated until the samples allowed stable measurements and calculation of the hydrodynamic radii. Standard deviations were calculated and considered as error estimates for the hydrodynamic radius of the molecule in solution.

### Analytical Size Exclusion Chromatography

Ligand binding to TGFR-3-ZP variants was studied in solution upon mixing the variants with equimolar amounts of TGF-β2 and using analytical SEC. After a 30 minutes-long incubation at 20°C, 30−50 µg of proteins and complexes were loaded onto a Superdex 200 10/300 GL column (GE Healthcare). Chromatography was performed at 4°C with a flow rate of 0.5 ml • min^-1^. Protein elution was monitored at 280 nm.

### Peptide Synthesis

Carboxy-terminus mimetic peptides (P1-mZP to P5-hZP, Fig.1) were synthesized by Fmoc/t-Bu-based solid phase synthesis using an automated multiple peptide synthesizer (SYRO from MultiSynTech, Witten, Germany), as described previously in detail [21]. The sequences were N-terminally acetylated (P1-mZP, P2-hZP, P3-mZP) and biotinylated (P4-mZP, P5-hZP), respectively, as well as C-terminally amidated. An additional lysine residue, whose side chain amino group was acylated with biotin, was attached to the C-terminus of P1-mZP, P2-hZP and P3-mZP. Cleaved peptides were purified by preparative HPLC, and their identities confirmed by ESI mass spectrometry.

### Surface Plasmon Resonance (SPR) Equilibrium Analyses

To analyze the interaction between TGFR-3-ZP and truncation variants thereof putative TGFR-3 ligands *i.e.* TGF-β2, BMP-2, Activin-A and GDF-5 were biotinylated using Sulfo-NHS-LC-biotin such that the ligands have 1 or 2 biotin moieties attached. A CM5 biosensor chip was activated using EDC/NHS according to manufacturer’s recommendation, coated with streptavidin to a density of about 3000 RU (1 RU  = 1 pg • mm^-2^), the ligands were subsequently immobilized via the biotin moiety on the Streptavidin biosensor chip as described before [22]. Sensorgrams for this interaction were recorded with a Biacore2000™ (GE Healthcare, Freiburg, Germany) at a flow rate of 10 µl • min^-1^ and 25°C using HBS500 buffer (10 mM HEPES, pH 7.4, 500 mM NaCl, 3.4 mM EDTA, 0.005% surfactant P20). The association and dissociation times were set to 300 s. After each data acquisition cycle the biosensor chips were regenerated with 4 M MgCl_2_ for 120 s. The data represent mean values of two independent experiments with at least six different analyte concentrations. Apparent binding affinities were determined from the dose dependency of equilibrium binding due to the fast association and dissociation rate constants.

Interactions between TGF-β2 and peptides derived from the C-terminal binding site of TGFR-3-ZP were acquired using a ProteOn XPR36 SPR system (Bio-Rad Laboratories, Munich, Germany). Because of the mass difference between TGF-β2 and the peptides, the peptides were immobilized onto the biosensor surface. Two different kinds of sensorchips were used, namely, either a commercially available neutravidin-coated sensorchip (Bio-Rad NLC chip) or neutravidin was coated on the surface of a GLC sensorchip using the same chemistry as above. The biotinylated peptides were then immobilized to the neutravidin coated matrices at densities between 300 and 600 RU. With the ProteOn SPR system, interaction sensorgrams were recorded at a flow rate of 200 µl • min^-1^ at 25°C using HBS150 buffer (10 mM HEPES, pH 7.4, 150 mM NaCl, 3.4 mM EDTA, 0.005% Tween 20). The association time was set to 120 s the dissociation time to 300 s, respectively. Affinities were derived by fitting the kinetic data using either a model considering a 1∶1 Langmuir type interaction with limited mass transfer or a simple 1∶1 Langmuir type interaction. The fitting model employing mass transfer limitation was necessary due to the high immobilization density of some peptides leading to fast rebinding effects during the dissociation phase.

### Crystal Structure Determination

Crystals of the ZP-C domain of mouse TGFR-3 were obtained under fortuitous circumstances with crystallization droplets containing TGFR-3-ZP and endoglycosidase F3 (Sigma-Aldrich). Prior to any crystallization trials TGFR-3-ZP (in 20 mM HEPES, pH 7.4, 20 mM NaCl, protein concentrations ranging from 10 to 35 mg • ml^-1^) was incubated for 30 minutes at room temperature with 75 ng endoglycosidase F3 per 100 µg TGFR-3-ZP. Protein droplets containing 0.2 µl reservoir solution and 0.2 µl of the above TGFR-3-ZP/endoglycosidase F3 mixture were equilibrated at 19°C using the sitting-drop vapor diffusion method over reservoir solution consisting of 0.2 M ammonium acetate, 0.1 M HEPES pH 6.0 to 7.0 and 20–30% (w/v) PEG 3350. Crystals grew within 2 to 8 weeks to final sizes of 20×20×50 µm^3^ and could not be further optimized (Fig. S1). Diffraction data were collected at PX beamline BL14.1 at Helmholtz Zentrum Berlin BESSY synchrotron facility [23]. 25% PEG 400 was added as cryo-protectant before the crystals were flash-cooled in liquid nitrogen. Diffraction data were processed using program XDS [24].

The structure could be solved by molecular replacement with program PHASER after the structure of rat TGFR-3-ZP-C (PDB accession code 3QW9) became available [13], [25]. The structure was refined with program PHENIX. During the initial rounds of crystallographic refinement, supplemental geometric restraints were derived from the search model and applied in program PHENIX [26]. Refinement was carried out to convergence until no further details could be interpreted. Data collection and refinement statistics are reported in Table 1.

**Table 1:** Data and refinement statistics of mouse TGFR-3-ZP-C.

Data statistics	
Wavelength (Å)	0.91841
X-ray source, detector	Bessy BL14.1, Rayonics MX-225 3×3 CCD
Space group	P2_1_2_1_2_1_
Unit cell parameters	a = 49.7 Å, b = 56.8 Å, c = 60.2 Å α = β = γ = 90°
Matthews Coefficient (Å^3^/Da^-1^)	2.24
No. molecules/ASU	1
Solvent content (%)	45.05
Resolution (Å)^+^	2.7 (2.77–2.7)
No. of reflections (Unique)	17,360 (4961)
Redundancy^+^	3.5 (3.6)
Completeness (%) ^+^	99.3 (100)
Mean *I*/(σ*I*)^ +^	11.2 (2.3)
*R*_sym_ (%) ^+^	10.2 (64.5)
Wilson B-factor (Å^2^)	52.1
**Refinement statistics**	
**Final ** ***R*** **-factor (%)**	
Working set	22.0
Working set+test set	22.4
Free *R*-factor (%) ^#^	29.6
**R.m.s. deviations**	
Bond lengths (Å)	0.017
Bond angles (°)	1.753
**Mean B-value (Å^2^)**	
Protein	50.0
Solvent	44.9
**Anisotropic scaling factors (Å^2^)**	
B11, B22, B33	−10.6, 21.8, −11.2
B12, B13, B23	0.0, 0.0, 0.0
No. of protein atoms	1,208
No. of solvent atoms	9
**Ramachandran plot ^##^**	
(%; preferred regions/allowed/outlier)	91.2/6.8/2.0

+ Numbers in parenthesis are for the highest-resolution shell. # 5% of reflections have been chosen as *R*_free_ set. *R*_sym_ is calculated as 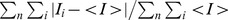 where *I_i_* is the *i^th^* observation of the n^th^ reflection and <*I*> the mean of all observations of the n^th^ reflection. ## Calculated with program COOT [35].

## Results

### Mouse TGFR-3-ZP Forms a Stable Domain in Solution

**Figure 2:**
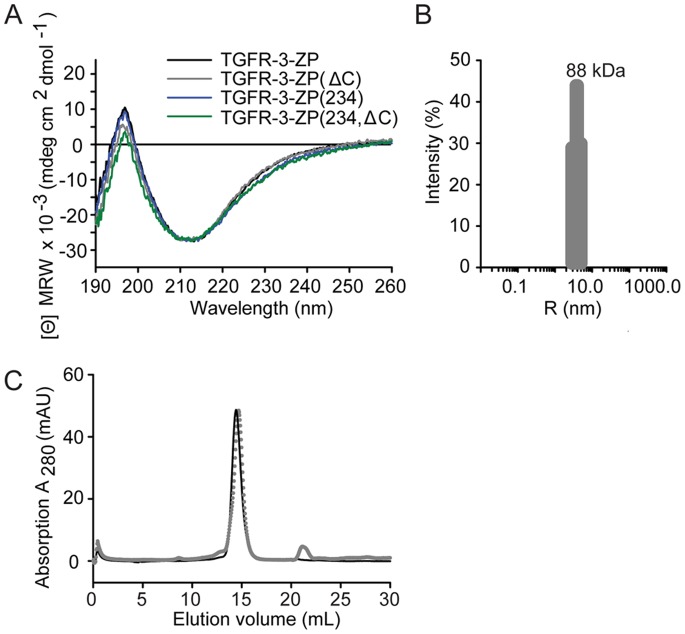
Secondary structure and oligomerization of TGFR-3-ZP proteins. (**A**) The CD spectra of all protein variants resemble spectra of properly folded proteins with predominantly antiparallel β-sheet structures. TGFR-3-ZP black, TGFR-3-ZP(ΔC) grey, TGFR-3-ZP(234) blue and TGFR-3-ZP(234,ΔC) green. (**B**) Monomodal size-distribution histogram of TGFR-3-ZP (2.0 mg⋅ml^−1^, at 20°C) monitored by dynamic light scattering. The intensity (in %) is plotted versus discrete particle sizes (*R*). In the histogram one peak with a hydrodynamic radius of 4.0 nm and a polydispersity of 20.9% is obtained. The apparent molecular weight of 88 kDa indicates a dimeric assembly of TGFR-3-ZP in solution. (**C**) Analytical size-exclusion chromatography of TGFR-3-ZP (black line) and TGFR-3-ZP(234) (grey dotted line) on a Superdex 200 10/300 column. The apparent molecular weight of the elution peak was calculated to be 75 kDa for TGFR-3-ZP and 70 kDa for TGFR-3-ZP(234), suggesting a dimeric assembly of TGFR-3-ZP.

Mouse TGFR-3-ZP (Fig. 1) was produced in HEK293 EBNA cells as a secretory glycoprotein with a monomer size of ∼44 kDa, as monitored by mass spectrometry (Fig. S2). The protein contains four potential *N*-glycosylation sites (positions 491, 570, 589 and 696). Whereas the asparagine residues at positions 570, 589 and 696 could be exchanged to glutamines to yield variant TGFR-3-ZP(234), mutation of Asn491 to glutamine abolished protein expression in HEK293 EBNA cells (data not shown). Circular dichroism **(**CD) spectra of TGFR-3-ZP and TGFR-3-ZP(234) show that both variants display similar folds (Fig. 2A). The spectra are in agreement with a predominantly antiparallel β-sheet structure as expected from previous CD experiments with uromodulin and as observed in the crystal structures of mouse ZP3-N, full-length chicken ZP3 and rat TGFR-3-ZP-C [11]–[13], [27], [28].

TGFR-3-ZP exhibits a monomodal size distribution in analytical dynamic light scattering (DLS) and on a size-exclusion chromatography (SEC) column (Fig. 2B, C) with apparent molecular weights of 88 kDa and 75 kDa, respectively. This hints that TGFR-3-ZP most likely forms a homodimer in solution. The TGFR-3-ZP(234) variant with one remaining potential *N*-glycosylation site migrates on SEC with a molecular weight of 70 kDa, again suggesting a dimeric assembly (Fig. 2C). Nonetheless, it has to be considered that the readout from DLS and SEC is influenced by the shape of the molecules and therefore do not provide absolute molecular weights. In this context it should be mentioned that in previous findings non-covalent dimers were only observed when the entire ectodomain of TGFR-3 was produced. Cleavage of the ectodomain into two halves with plasmin generated fragments that migrate as monomers in SEC experiments [6].

### TGF-β2 Binding Protects TGFR-3-ZP against Proteolytic Removal of the C-terminal EHP

**Figure 3:**
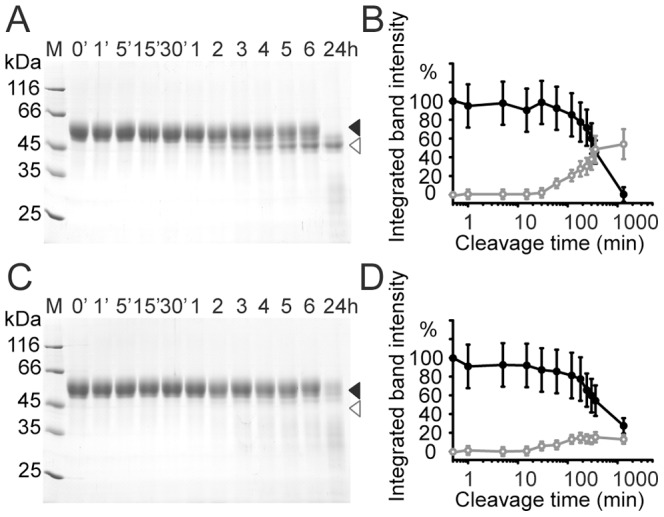
Binding of TGF-β2 protects TGFR-3-ZP against proteolysis. (**A**) Limited proteolysis of recombinant murine TGFR-3-ZP in the absence of TGF-β2 using proteinase K and monitored by SDS-PAGE. TGFR-3-ZP is marked with a black triangle and the emerging TGFR-3-ZP(ΔC) fragment with a grey triangle. (**B**) Limited proteolysis experiment as shown in (A) but repeated in the presence of TGF-β2.

Digestion of TGFR-3-ZP in the absence of its ligand TGF-β2 with limiting concentrations of the unspecific serine protease proteinase K yields one major fragment with a modest molecular mass reduction of about 5.5 kDa as monitored by mass spectrometry and SDS-PAGE (Fig. 3A, S2, [19]). At longer digestion times this fragment becomes completely degraded without the emergence of any additional discrete cleavage products. N-terminal Edman sequencing shows that in this fragment only the His-tag is cleaved off with no further truncation at the N-terminus (Fig. S2). The molecular mass reduction in this fragment (termed TGFR-3-ZP(ΔC) from hereon) must therefore originate from a truncation towards the C-terminus of TGFR-3-ZP at approximately position 735 (±5 residues). When mapping this position onto known ZP domain structures, the cleavage site maps within the FG loop that extends from the ZP core domain and precedes the EHP sequence (see below) [8], [11], [13]. Therefore the new C-terminus of the fragment TGFR-3-ZP(ΔC) obtained by limited proteolysis can be considered to approximately correspond to that of the ZP core domain of murine TGFR-3 (Fig. 1). The proteolysis experiment also hints that in contrast to other ZP domains, the ZP core domain of murine TGFR-3-ZP displays a relatively rigid fold, which cannot readily be cleaved into two structurally independent ZP-N and ZP-C subdomains (Fig. 3A) [8], [13]. A similar behaviour of TGFR-3 has previously also been reported with plasmin and similar results are also obtained with the protease subtilisin (Fig. S2C) [6].

The proteolytically truncated proteins TGFR-3-ZP(ΔC) and TGFR-3-ZP(234,ΔC) were produced in milligram amounts and after further purification investigated with CD spectroscopy (Fig. 2A). The CD spectra of the truncated proteins are very similar to those of the non-truncated proteins. A difference spectrum in which the CD spectrum of TGFR-3-ZP(ΔC) is subtracted from that of TGFR-3-ZP suggests that predominantly regions devoid of defined secondary structure elements are cleaved off in TGFR-3-ZP(ΔC) (Fig. S3).

Unexpectedly, a different behaviour is observed when repeating the limited proteolysis experiments with TGFR-3-ZP in the presence of the ligand TGF-β2. When TGF-β2 is present in the proteolysis setup then the digestion rate is considerably reduced and most importantly almost no TGFR-3-ZP(ΔC) is formed (Fig. 3B, S4). This observation provides first evidence that the C-terminal region of TGFR-3-ZP participates in ligand binding.

### The C-terminal Region of TGFR-3-ZP Harbours a Major Ligand-binding Site

**Figure 4:**
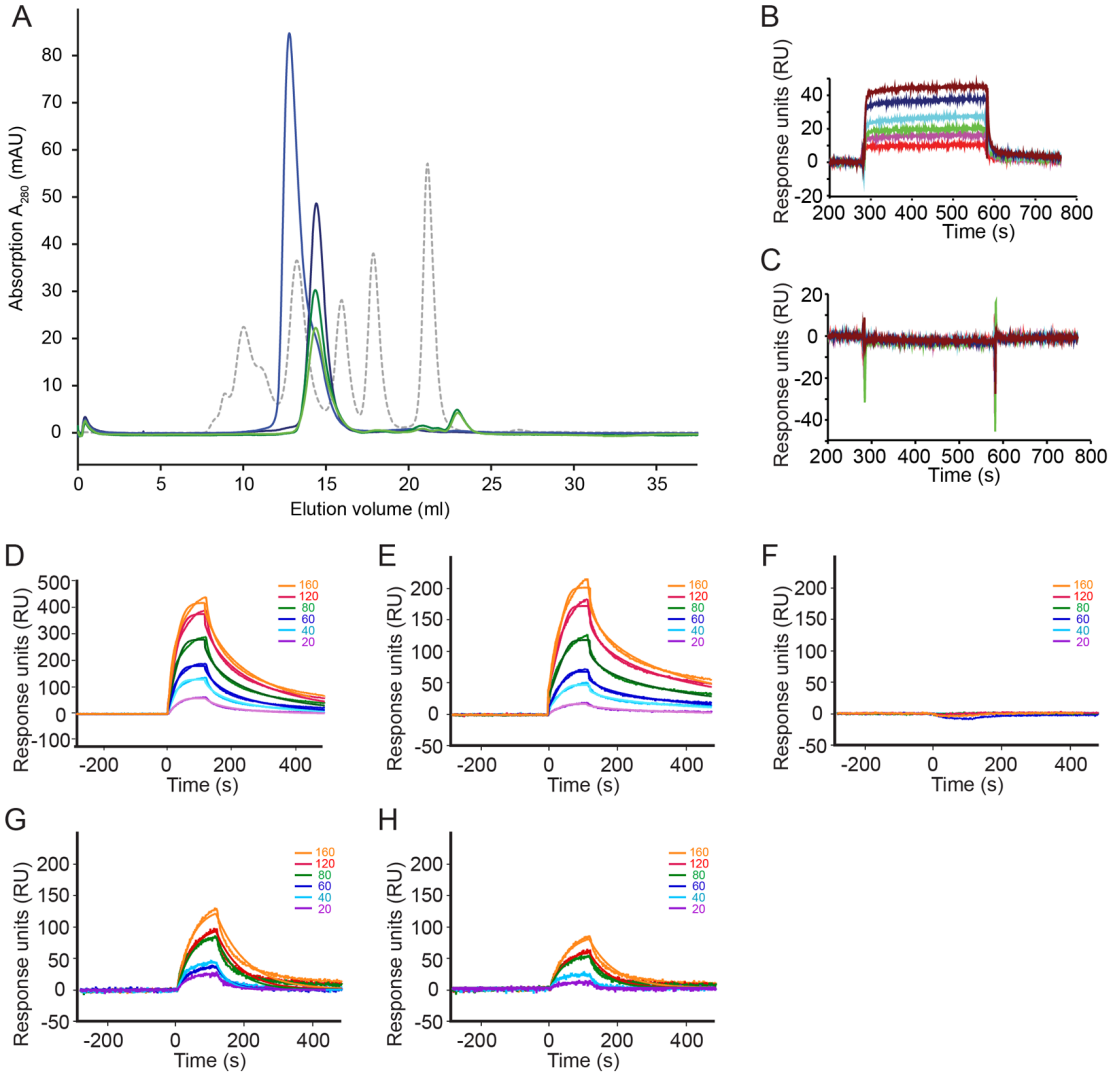
Analysis of the TGF-β2-binding activity of TGFR-3-ZP variants and peptides. (**A**) Analytical size-exclusion chromatography of TGFR-3-ZP in the presence (blue) and absence of TGF-β2 (deep blue). The migration behaviour of TGFR-3-ZP is also compared to that of the truncated protein TGFR-3-ZP(ΔC) either with (green) or without TGF-β2 (dark green). In absence of TGF-β2, TGFR-3-ZP and TGFR-3-ZP(ΔC) show similar retention times. The absorbance spectra of the samples are shown together with the elution profile of molecular weight standards (grey dotted curve, major peaks from left to right: ferritin (440 kDa), aldolase (158 kDa), BSA (67 kDa), ovalbumin (43 kDa) and myoglobin (17 kDa)). *K_av_*-values derived from peak maxima are listed in supplementary Fig. S5. (**B** *) In vitro* interaction analysis of TGFR-3-ZP binding with TGF-β2 immobilized on a biosensor surface using SPR. The analyte TGFR-3-ZP was injected at time point 300 s, six different analyte (TGFR-3ZP) concentrations (indicated by different colors) were used. At time point 600 s buffer was perfused to record dissociation of TGFR-3-ZP from the TGF-β2 biosensor surface. (**C**) Same as (B) but using TGFR-3-ZP(ΔC) as analyte. No TGF-β2 binding activity is observed for the truncated mutant TGFR-3-ZP(ΔC). (**D**) to (**H**) Sensorgrams obtained with peptides mimicking the C-terminus of human and mouse TGFR-3-ZP immobilized onto a neutravidin-coated biosensor via an N- or C-terminal chemically attached biotin moiety and using TGF-β2 as analyte. (**D**) Interaction TGF-β2 with immobilized P1-mZP comprising residues 735–782. Six different analyte concentrations (indicated by color and numbers) were used for analysis. (**E**) Same as in (D) but for the peptide P2-hZP comprising residues 737–788 of human TGFR-3. (**F**) Same as in (D) but for peptide P3-mZP (residues 749–782). P3-mZP does not show any TGF-β2 binding. (**G**) Sensorgram for the interaction of TGF-β2 (as analyte, five different concentrations were used) and P4-mZP comprising residues 732–748. (**H**) Same as in (G) but for the peptide P5-hZP comprising residues 734–750. The fit of the SPR data is indicated by a solid line.

In order to further resolve the role of the C-terminal region of TGFR-3-ZP in ligand binding, we incubated samples of murine TGFR-3-ZP and TGFR-3-ZP(ΔC) with purified human TGF-β2 at equimolar ratios for 30 min and analysed complex formation by analytical SEC. Ligand-free TGFR-3-ZP elutes as a single homogeneous peak. TGFR-3-ZP incubated with TGF-β2 elutes at a significant earlier volume in agreement with an anticipated increased molecular weight for a TGFR-3-ZP - TGF-β2 complex (Fig. 4A, S5). Provided that the elution peak observed for the sole TGFR-3-ZP sample accounts for a molecular dimer, the composition of the receptor-ligand complex can be estimated from the observed retention volume as corresponding to either a 4∶2 or possibly a 4∶4 stoichiometry, meaning that two TGFR-3-ZP dimers interact with either one or two TGF-β2 dimers.

In contrast to TGFR-3-ZP, no peak shift is observed for TGFR-3-ZP(ΔC) upon addition of TGF-β2. This shows that the C-terminally truncated variant TGFR-3-ZP(ΔC) lost its ability to bind TGF-β2 (Fig. 4A, S5). However, TGFR-3-ZP(ΔC) did not lose its ability to form homodimers since it still elutes at a similar retention volume as TGFR-3-ZP. When repeating these experiments with TGFR-3-ZP(234) an identical behaviour is obtained (data not shown). In agreement with previous reports, deletion of three *N*-glycosylation sites in TGFR-3-ZP(234) does not alter ligand binding in TGFR-3-ZP [5], [29].

In order to quantify the ligand-binding affinities of the TGFR-3-ZP variants, we performed SPR equilibrium analyses with TGF-β2 and related ligands immobilized on the chip surface [30]. Binding affinities between the TGFR-3-ZP domain and the selected TGF-β members were measured by immobilizing biotinylated TGF-β ligands onto a streptavidin coated biosensor surface. TGFR-3-ZP, the glycosylation-attenuated TGFR-3-ZP variant as well as the C-terminally truncated TGFR-3-ZP variant were perfused over this biosensor as analytes. The dissociation constants (*K*_D_) were obtained by fitting the equilibrium response as a function of injected receptor concentration to a standard binding isotherm ranging from 2.4 µM for TGFR-3-ZP to 2.7 µM TGFR-3-ZP(234) and are in good agreement with previous reports for the soluble membrane-proximal C-terminal half of the TGFR-3 ectodomain with binding affinities of 2.6 µM for TGF-β2, 5.8 µM for TGF-β3 and 9.7 µM for TGF-β1 (Fig. 4B, Table 2) [6]. The SPR measurements also validate the use of human TGF-β2 for studying ligand-binding to murine TGFR-3-ZP. In fact, human and murine TGF-β2 only differ in three amino acid residues, namely Ser361Thr (human *versus* mouse sequence), Arg362Lys and Lys396Asp.

**Table 2:** Binding affinities determined by SPR from the interaction of TGFR-3 and variants thereof with immobilized TGF-β ligands TGF-β2 and BMP-2.

TGFR-3 variant	Residues	TGF-β2	BMP-2
TGFR-3-ZP	438–782	2.4 µM	29.1 µM
TGFR-3-ZP(234)	438–782	2.7 µM	34.6 µM
TGFR-3-ZP(ΔC)	438–734	NB*	NB
TGFR-3-ZP(234, ΔC)	438–734	NB	NB

* NB no detectable binding.

More importantly however, and consistent with our SEC experiments, the SPR measurements show that the proteolytically truncated proteins TGFR-3-ZP(ΔC) and TGFR-3-ZP(234,ΔC) do not display any TGF-β2 binding (Fig. 4C, Table 2). We also probed binding of human BMP-2 by SPR with BMP-2 immobilized on the chip surface. The *K*_D_ values obtained for BMP-2 binding to TGFR-3-ZP and TGFR-3-ZP(234) are about one order of magnitude lower than those determined for TGF-β2 (29.1 µM and 34.6 µM, respectively; Fig. S6A, Table 2). As for TGF-β2, no binding was observed for the interaction of BMP-2 with the truncated proteins TGFR-3-ZP(ΔC) and TGFR-3-ZP(234,ΔC) (Fig. S6B, Table 2). Human GDF-5 and Activin-A do not bind to the TGFR-3 protein used in this study (Fig. S6C and data not shown).

### Peptides Covering the C-terminal Region of TGFR-3-ZP Bind to TGF-β2

Truncation of the C-terminus at around position 735 of TGFR-3-ZP abolishes ligand binding. In order to obtain a positive readout and to further narrow down the ligand-binding site, we synthesized five peptides that span different segments of the truncated C-terminus in TGFR-3-ZP(ΔC) (Fig. 1). Two peptides displaying either the murine or human TGFR-3 sequence, namely P1-mZP and P2-hZP, cover the entire truncated C-terminus and span approximately from the end of the bioinformatically defined ZP core domain (position 728, [9]) to the beginning of the transmembrane domain of TGFR-3 (position 782, Fig. 1). In the additional peptides P3-mZP, P4-mZP and P5-hZP this segment is subdivided into two parts, namely into a more extended C-terminal part (P3-mZP) and a shorter N-terminal part (P4-mZP and P5-hZP, Fig. 1).

Due to the small molecular masses of the peptides (in comparison to TGF-β2) we changed our SPR setup such that the peptides were immobilized onto a neutravidin-coated biosensor *via* a biotin moiety. In this altered setup with the peptides immobilized, the binding affinities to the dimeric TGF-β2 are expected to be affected by avidity effects. This can be explained by the fact that due to the density of the peptides on the biosensor surface the dimeric TGF-β2 can bind simultaneously to two peptides and we thus refer to this as a 1∶2 interaction model [22]. During the dissociation phase release of the TGF-β2 from the biosensor surface therefore requires simultaneous breaking of both interactions, which according to statistical thermodynamics is less likely [31]. Thus, we expect this setup to generate slower dissociation rates and hence higher affinities.

The SPR measurements show that the two long peptides P1-mZP and P2-hZP indeed bind to human TGF-β2 with affinities of 8 nM and 15 nM, respectively (Fig. 4D, E, Table 3). In contrast to this, the shortened mouse peptide P3-mZP, which lacks the first 14 N-terminal residues present in the peptides P1-mZP and P2-hZP shows no binding at all (Fig. 4F). To investigate the importance of the 14 N-terminal residues further, we repeated the SPR measurements employing two peptides comprising just these N-terminal residues, namely P4-mZP and P5-hZP. However, in contrast to the previous peptides where the biotin moiety was attached to the C-terminus of the peptides, both 17mer peptides P4-mZP (TGFR-3 residues 732 to 748) and P5-hZP (residues 734 to 750) were immobilized to the biosensor via an N-terminally attached biotin moiety. Measurement of the binding affinities yielded equilibrium binding constants *K*_D_ of about 190 nM and 350 nM for P4-mZP and P5-hZP, respectively, and confirm that the major part of the binding epitope of the ZP domain for TGF-β2 is constrained to the residues displayed in these peptides (Fig. 4G, H, Table 3). The 24 and 50-fold lower affinities of these peptides in comparison to the peptides that cover the entire C-terminus might be due by several effects. Possibly, the accessibility of the binding determinants might change when the peptides are attached to the biosensor via an N-terminally or C-terminally linked biotin moiety. It is also possible that the C-terminal residues of P1-mZP and P2-hZP contribute to the binding of TGF-β2 either directly or indirectly by altering the conformation of those residues that form the binding determinant. A comparison of the association rate constants for the long (P1-mZP and P2-hZP) and the short (P4-mZP and P5-hZP) peptides shows that the longer peptides exhibit an about 40- to 60-fold faster association rate, with the dissociation rates being almost identical (differing by 1.4 fold or less). Whereas the latter are a measure of the complex stability indicating that the same non-covalent interactions can be formed for the long and short peptide, the increased association rates point to the shorter peptides possibly undergoing a conformational change required for TGF-β2 binding. Importantly, however, these experiments clearly confirm the importance of the C-terminal residues of TGFR-3-ZP for TGF-β2-binding and narrow down the ligand-binding site to about 14 residues.

**Table 3:** Binding affinities determined by SPR from the interaction of immobilized peptides mimicking the TGFR-3 C-terminus and the TGF-β ligand TGF-β2.

peptides mimicking TGFR-3	Residues		TGF-β2	
		*K*_D_ * [nM]	*k*_on_ (×10^5^) [M^−1^s^−1^]	*k*_off_ (×10^−2^) [s^−1^]
P1-mZP	735–782	7.9±6.1	52±4.3	2.9±0.39
P2-hZP	737–788	15±6.7	44±39	4.4±2.6
P3-mZP	749–782	NB	–	–
P4-mZP	732–748	192±64.1	1.1±0.4	2.0±0.70
P5-hZP	734–750	353±113	0.79±0.27	2.9±1.8

* derived from *K*_D_ = *k*_off_/*k*_on_; ND not determined; NB no measureable binding.

### Crystal Structure of TGFR-3-ZP-C

**Figure 5:**
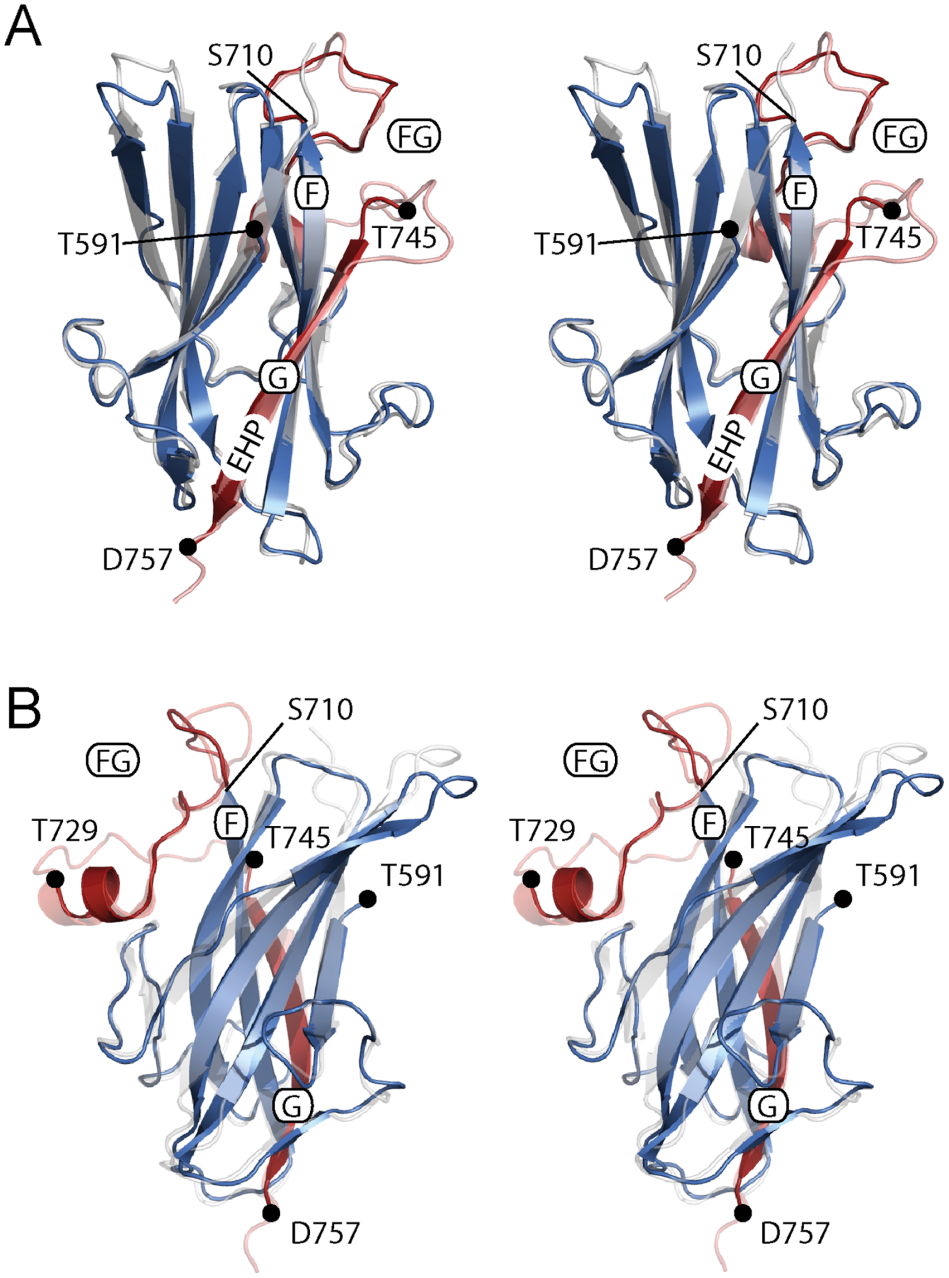
Crystal structure of murine TGFR-3-ZP-C. (**A**) Stereographic ribbon representation of the murine TGFR-3-ZP-C structure (in blue and red). The ZP-C domain in the crystals extends from Thr591 to Asp757. No density is visible for residues 730 to 744. The FG loop connecting β-strands F to G (residues 711 to 746) and β-strand G are shown in red. The EHP segment that is part of β-strand G is marked. For comparison reason the structure of rat ZP-C (PDB entry 3QW9, [13]) that was used to solve the structure of murine ZP-C is shown in transparent grey and light red. In contrast to the murine ZP-C structure, the entire FG loop is visible in the rat ZP-C structure. Chain breaks in the murine ZP-C structure are marked with black dots. (**B**) Stereographic representation of the rat and murine ZP-C structure identical to that in panel (A) but after application of an approximately 110° rotation around a vertical axis.

The mouse TGFR-3-ZP protein was crystallized in the presence of the endoglycosidase F3 under the assumption that *in situ* deglycosylation facilitates crystallization. Surprisingly, the structure determination revealed that the crystals did not contain the entire TGFR-3-ZP domain but only a fragment that corresponds to the ZP-C subdomain (Figs. 5 and S1). This breakup was unexpected considering that we did not see a cleavage of murine TGFR-3-ZP into a ZP-N and ZP-C fragment in the limited proteolysis experiments with proteinase K and subtilisin (Figs. 3 and S2). It presently remains unclear why incubation with endoglycosidase F3 lead to ZP-C formation.

The crystals of the murine ZP-C diffracted to about 2.7 Å and the structure was refined to *R*-values of 22.0% and 29.6% for *R*_work_ and *R*_free_, respectively (Table 1). The polypeptide chain could be traced contiguously from residues 591 to 757 with the exception of a stretch of 11 residues that is part of the FG loop (residues 730 to 744, Fig. 5). Since the crystals grew upon *in situ* proteolytic cleavage, the exact termini of the crystallized fragment remain uncertain. Tight crystal packing contacts around residue 591 preclude the presence of any residues preceding residue 591 such as for example residue Asn589, a potential N-glycosylation site. In contrast, space is available in the crystals for residues that would follow residue 757. In case of the bridging residues 730 to 744 in the FG loop we observe residual, however non-interpretable, difference density hinting that this segment remained largely intact in the crystals and that the lack of defined electron density is caused by conformational disorder in this segment.

As previously seen in rat TGFR-3 and other ZP domain containing proteins, the overall fold topology is reminiscent of the immunoglobulin superfold and comprises a β-sheet sandwich formed by two four-stranded β-sheets (strands A, B, E, D and C’, C, F, G) [32]. As noted before, the intervening loop segments (termed AB, BC, CC’, …) differ considerably in size and in case of loop FG also contains a small α-helical segment termed αFG [11]–[13].With the exception of strand G (residues 745 to 757 in murine TGFR-3, see below) the structure compares well to the corresponding rat ZP-C fragment, which was used as a search model during molecular replacement ([13], Fig. 5). Overall, the root mean square (r.m.s.) deviation between common main chain atoms in the rat and mouse structure is 0.7 Å.

Unexpectedly, the amino acid registration of strand G is shifted by four residues in murine ZP-C when compared to the corresponding segment in rat ZP-C (Fig. S7) [13]. This shift in registration became obvious during the final refinement stages. Negative density was observed for a phenylalanine and lysine side chain and positive density at the position of an alanine side chain. Shifting the sequence by four residues caused a drop in the *R*-values of about 1 to 2% and replaced the phenylalanine by leucine, lysine by valine and alanine by glutamine. Other residues did either not change their identity or were replaced by residues with similar sizes such as threonine by proline or valine. A close inspection of the corresponding electron density in the 2.0 Å resolution rat ZP-C crystal structure does not provide any indications for model building ambiguities in this structure (Protein Data Bank (PDB) entry 3QW9, [13]). We therefore conclude that the shift in the registration of strand G, which contains the EHP sequence, constitutes a genuine difference between both structures. The alternative registration of strand G in rat and murine ZP might be a direct consequence of the expected more loose interaction between the EHP and the remaining ZP-C domain since transient interactions between the EHP segment and the ZP core domain have been proposed to be at the heart of a mechanism that regulates polymerization in many ZP domain-containing proteins [8], [10], [15].

## Discussion

The identification of the binding site for TGF-β-like growth factors in TGFR-3 has been the subject of numerous mutagenesis studies [3]–[7], [33], [34]. Deletion studies not only identified two separate binding sites in the N- and C-terminal half of the extracellular portion of TGFR-3 but single site mutagenesis studies also proposed a set of individual residues as important binding determinants [7]. Important additional insight has recently been gained by a crystal structure determination which allowed mapping these residues onto the crystal structure of the ZP-C subdomain of rat TGFR-3 [13].

In the present study we used a different approach and identified a novel major binding site for TGF-β-like growth factors in TGFR-3. Using a set of *in vitro* experiments such as limited proteolysis, size exclusion chromatography, synthetic peptides and SPR measurements we could show that the C-terminal region of the ZP-domain of TGFR-3 displays a high affinity-binding site for TGF-β2 and BMP-2. Removal of residues C-terminal to position 735 in murine TGFR-3-ZP through proteolytic cleavage abolishes ligand-binding. Conversely the presence of TGF-β2 protects the C-terminal residues from proteolytic cleavage. In order to resolve any ambiguities that might be associated with the exact position of the proteolytic cleavage site we synthesized a set of peptides that cover the C-terminal part of TGFR-3-ZP, namely a peptide P1-mZP that covers residues 735 to 782 and P3-mZP that spans residues 749 to 782. Whereas P1-mZP and the homologous human sequence (peptide P2-mZP) bind TGF-β2 with high affinity, the peptide P3-mZP did not show any binding in our SPR measurements. These experiments suggest the TGF-β2 binding site to be located within residues 735 to 749 of the murine TGFR-3. This hypothesis could be confirmed by using small 17mer peptides consisting only of residues 732 to 749 (P4-mZP) and 737 to 750 (P5-hZP) of murine and human TGFR-3, which both bind TGF-β2 with affinities only one order of magnitude lower than the peptides mimicking the complete C-terminus of TGFR-3.

We also succeeded in determining the crystal structure of the murine ZP-C domain (residues 591 to 757 of TGFR-3-ZP). With a single difference, regarding the registration of the EHP sequence, this structure is highly similar to the structure of previously determined rat ZP-C [13]. In these structures, the proteolytic truncation site at around residue 735 that we identified in TGFR-3-ZP(ΔC) and that abolishes ligand binding maps within the loop that connects β-strand F to β-strand G (FG-loop, Fig. 5). Proteolytic cleavage appears to occur shortly after a small α-helical segment termed αFG contained within the FG loop [11]–[13]. Peptides that we identified as containing major ligand-binding determinants map to the TGFR-3-ZP segment and their sequences extend from the end of αFG to the beginning of β-strand G. The crystal structure shows that these residues appear especially well suited for ligand binding since these residues form a surface exposed loop that should be readily accessible for ligand-binding. The FG loop is not resolved in the murine ZP-C crystal structure due to conformational flexibility. The loop is however well resolved in the crystal structure of rat ZP-C [13]. Its composition is rather unusual for a surface exposed loop since it contains a number of hydrophobic residues including one tryptophan and two methionines (Fig. 1). Further investigations will be needed to establish whether this contiguous peptide provides for novel opportunities for modulating TGF-β signaling.

Interestingly, segment 730 to 744 does not cover those regions of the ZP domain that have been identified as important for TGF-β2 and inhibin binding in single site mutagenesis studies before [7]. Previously highlighted residues are part of the segments 606 to 618 and 633 to 635 of murine TGFR-3-ZP. These map either onto the AB-loop or onto strand C of ZP-C [7], [13]. As already discussed for the crystal structure of rat ZP-C the most important residues in these segments such as for example Val612 (murine numbering) are not surface exposed and are oriented towards the interior of the ZP domain [13]. Since mutants such as Val612Tyr have so far only be studied in cell-based assays it is possible that these mutations only indirectly abrogate ligand binding possibly through a destabilization of the ZP-C domain [7], [13]. In contrast, the proteolytically C-terminally truncated and ligand-binding deficient variant TGFR-3-ZP(ΔC) studied here, displayed a CD spectrum that corresponds to a well folded protein highly similar to that of non-truncated TGFR-3-ZP (Fig. 2A).

Additional investigations are needed for better understanding the quaternary structure of TGFR-3. This also extends to the structure of the ligand receptor complex. Our size exclusion chromatography experiments hint that a high molecular weight complex is formed with possibly a 4∶2 or even 4∶4 stoichiometry, meaning that two TGFR-3-ZP dimers interact with either one or two TGF-β2 dimers. It presently remains unclear how such a complex would be topologically organized, and whether all four ZP-C FG loops present in such a complex would participate in identical ligand interactions. This would obviously only be possible in a 4 to 4 complex.

Our findings potentially provide novel experimental insight into the general function of ZP domain-containing proteins. The comparison of the crystal structures of rat and murine ZP-C, and, more precisely, the alternative sequence registration in strand G, provides structural evidence that the EHP segment that follows the FG-loop only loosely associates with the ZP core domain. This observation supports mechanisms in which removal and/or the exchange of the EHP segment controls the polymerization of ZP domain-containing proteins [8]. It is quite tempting to speculate that association and dissociation of the EHP segment could also play a role in TGF-β2 presentation by TGFR-3 and facilitate TGF-β2 transfer to TGFR-1 and TGFR-2. Taken together, our studies suggest that a similar region, FG-loop plus EHP segment, might be important for the controlled polymerization of ZP domain-containing proteins and high affinity growth factor recognition in TGFR-3.

The atomic coordinates and structure factors of the ZP-C domain of mouse TGFR-3 have been deposited in the Protein Data Bank (code 4AJV).

## Supporting Information

Figure S1 **Murine TGFR3-ZP-C crystals analyzed by SDS-PAGE.** Individual crystals were washed repeatedly in reservoir solution, pooled, solubilized in SDS sample buffer and boiled before loading onto an SDS polyacrylamide gel.
(TIF)

Figure S2 **Proteinase treatment of recombinant TGFR-3-ZP produces a stable fragment. (**A) Mass spectrometry analysis of TGFR-3-ZP before (TGFR-3-ZP) and after limited digestion with proteinase K (TGFR-3-ZP(ΔC)). In this experiment thrombin-treated and purified protein without His-tag was split into two aliquots. One aliquot (TGFR-3-ZP) was directly analyzed by mass spectrometry whereas the second aliquot was used for a preparative proteinase K digestion. The proteinase K-cleaved protein was purified by an additional gel filtration step and then analyzed by mass spectrometry (TGFR-3-ZP(ΔC)). Peaks are marked with their corresponding molecular mass. The mass difference between these two proteins is about 5.5 kDa. (**B**) N-terminal sequencing of TGFR-3-ZP(ΔC). Upper sequence: N-terminal sequence of the His-tagged TGFR-3-ZP construct. The position where Thrombin is expected to cleave is marked with a black triangle. Lower lines: N-terminal sequence of TGFR-3-ZP(ΔC) as obtained by Edman sequencing. Please note that in this case, the limited digestion with proteinase K was performed using His-tagged TGFR-3-ZP. N-terminal sequencing showed that, with the exception of the N-terminal histidines, no further truncation occurred at the N-terminus. Therefore the 5.5 kDa mass reduction observed for TGFR-3-ZP(ΔC) in the mass spectrometry analysis results from a C-terminal truncation of TGFR-3-ZP. At two positions, the amino acids could not be identified unambiguously by Edman sequencing (X = any amino acid). (**C**) Digestion of recombinant His-tagged TGFR-3-ZP protein with limiting concentrations of the unspecific serine protease subtilisin instead of proteinase K. Samples were retrieved at indicated time intervals and analyzed with SDS-PAGE. As for proteinase K, subtilisin treatment of TGFR-3-ZP generates one major proteolysis resistant fragment (black triangle). This fragment is highly similar to the fragment obtained after digestion with proteinase K (Fig. 3).
(TIF)

Figure S3 **Circular dichroism difference spectra of TGFR-3 variants.** CD difference spectra were obtained by subtracting the spectra of TGFR-3-ZP(ΔC) and TGFR-3-ZP(234,ΔC from the spectra of TGFR-3-ZP and TGFR-3-ZP(234), respectively. The difference spectra resemble the CD spectrum of a protein devoid of secondary structure elements. This possibly hints that the C-terminally cleaved-off fragments are largely disordered.
(TIF)

Figure S4 **Video-densitometric quantification of protein bands in SDS gels.** (**A**) Band intensities obtained in the SDS gels after limited proteolysis of TGFR-3-ZP with proteinase K (see Fig. 3A, main text) were analyzed by video-densitometry. (**B**) Same as for (A) but referring to the sample that was incubated with TGF-β2 prior to protease exposure (see Fig. 3C, main text). Full-length TGFR-3-ZP protein, black line; proteolytic fragment TGFR-3-ZP core, grey line.
(TIF)

Figure S5 **Molecular weight calibration curve used for the estimation of the oligomeric state of various protein samples.** The calibration curve was derived from the elution profile of reference proteins (Fig. 4A, main text). The standard proteins’ partitition coefficients (*K_av_*) are plotted against the specific molecular weights. The *K_av_*-values determined for TGFR-3-ZP and TGFR-3-ZP(ΔC) are marked for the free forms and the samples that have been incubated with TGF-β2. Colours are as in Fig. 4A (main text).
(TIF)

Figure S6 **SPR measurements with TGFR-3-ZP and TGFR-3-ZP(ΔC).** (**A**) TGFR-3-ZP and immobilized BMP-2, (**B**) TGFR-3-ZP(ΔC) and immobilized BMP-2 and (**C**) TGFR-3-ZP and immobilized GDF-5.
(TIF)

Figure S7 **Shift of β-strand G registration and of the EHP sequence in the murine TGFR-3-ZP-C structure when compared to the structure of rat TGFR-3-ZP-C.** (**A**) Structural alignment of the EHP of rat (upper line) and murine TGFR-3-ZP-C (lower line). Whereas the overall sequence identity between rat and murine TGFR-3-ZP (residues 590 to 755, murine numbering) is 97%, the identity is 100% in the displayed segment (middle line). Hence, the sequence assignment in this segment in the first murine ZP-C model was kept identical to that in the rat ZP-C structure (PDB entry entry 3QW9). However, in subsequent rounds of crystallographic refinement it became obvious that the sequence in the murine ZP-structure has to be offset by four residues when compared to the rat structure (see also panels B to F). Although the sequence identity of the structurally aligned sequences is now as low as 23%, the sequence similarity is still in the order of 40% (as reported by the SIAS server, http://imed.med.ucm.es/Tools/sias.html). In lower letters, amino acid residues not visible in the electron density maps of either the murine or rat ZP-C structure. In bold, residues forming the extended strand G in ZP-C, which includes the EHP sequence. (**B** and **C**) 2mFo-DFc and mFo-DFc electron density in strand G in the murine ZP-C structure when using an identical amino acid registration in murine and rat TGFR-3-ZP-C. This registration leads to negative difference density at the position of the side chain of Phe746 (panel B) and unexplained positive electron density near residue Ala751 (panel C). The 2mFo-DFc electron density (in blue) is displayed at a 1.0 σ level in all panels. The positive and negative difference electron density of the mFo-DFc electron density map in this and subsequent panels is displayed at 3.0 (in green) and -3.0 sigma levels (in red), respectively. (**D** and **E**) A four residue shift in the registration of the protein sequence explains the 2mFo-DFc and mFo-Fc electron density in this segment better than the initial registration shown in panels (B and C). This leads to the replacement of residues Phe746, Lys748 and Ala751 with residues Leu750, Val752 (panel D) and Gln755 (panel E), respectively. (**F** and **G**) 2mFo-DFc (in blue) and mFo-Fc electron density (in red and green) in this segment after a round of crystallographic refinement with the correct registration. The shift in the registration improved both *R_work_* (21.9 *versus* 23.1%) and *R_free_* (32.0 *versus* 33.7%) by approximately 1 to 2%.
(TIF)
