# Supplementary figures and images for: Identification of a Novel TGF-β-Binding Site in the Zona Pellucida C-terminal (ZP-C) Domain of TGF-β-Receptor-3 (TGFR-3)
Source: PLoS One. 2013 Jun 27;8(6):e67214. doi: 10.1371/journal.pone.0067214 (PMC3695229; doi:10.1371/journal.pone.0067214)

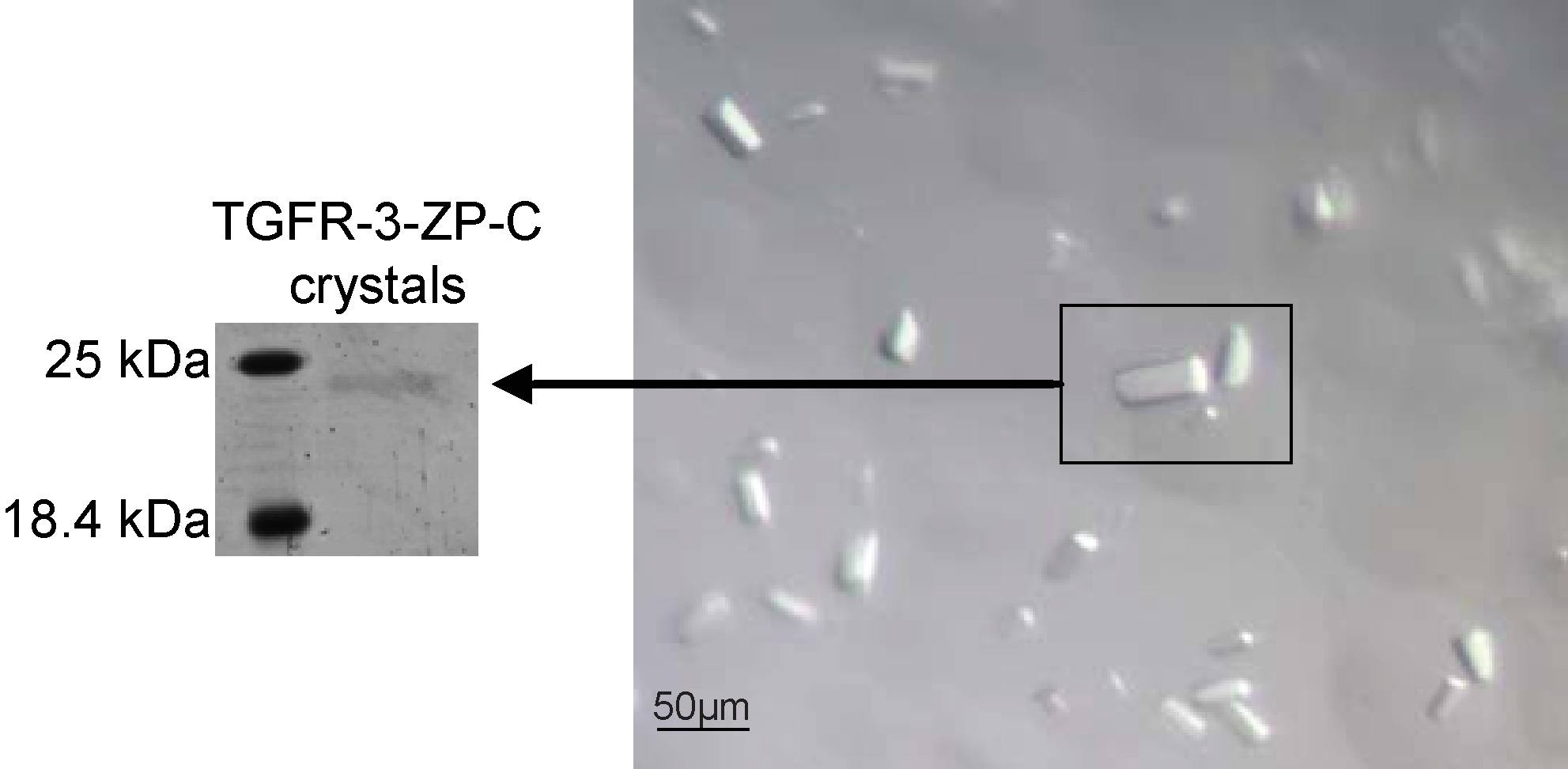

Supplement: Figure S1 — Murine TGFR3-ZP-C crystals analyzed by SDS-PAGE. Individual crystals were washed repeatedly in reservoir solution, pooled, solubilized in SDS sample buffer and boiled before loading onto an SDS polyacrylamide gel. (TIF) [file pone.0067214.s001.tif]

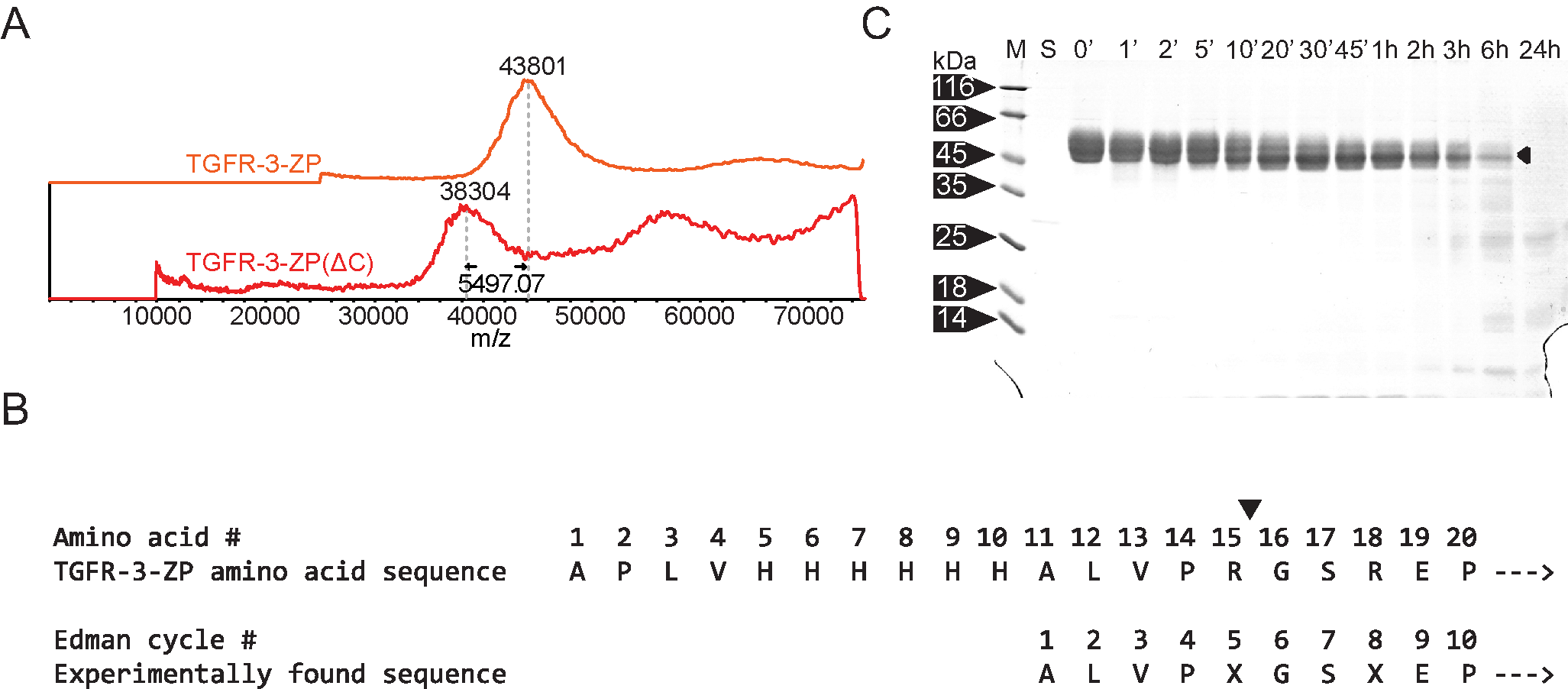

Supplement: Figure S2 — Proteinase treatment of recombinant TGFR-3-ZP produces a stable fragment. (A) Mass spectrometry analysis of TGFR-3-ZP before (TGFR-3-ZP) and after limited digestion with proteinase K (TGFR-3-ZP(ΔC)). In this experiment thrombin-treated and purified protein without His-tag was split into two aliquots. One aliquot (TGFR-3-ZP) was directly analyzed by mass spectrometry whereas the second aliquot was used for a preparative proteinase K digestion. The proteinase K-cleaved protein was purified by an additional gel filtration step and then analyzed by mass spectrometry (TGFR-3-ZP(ΔC)). Peaks are marked with their corresponding molecular mass. The mass difference between these two proteins is about 5.5 kDa. (B) N-terminal sequencing of TGFR-3-ZP(ΔC). Upper sequence: N-terminal sequence of the His-tagged TGFR-3-ZP construct. The position where Thrombin is expected to cleave is marked with a black triangle. Lower lines: N-terminal sequence of TGFR-3-ZP(ΔC) as obtained by Edman sequencing. Please note that in this case, the limited digestion with proteinase K was performed using His-tagged TGFR-3-ZP. N-terminal sequencing showed that, with the exception of the N-terminal histidines, no further truncation occurred at the N-terminus. Therefore the 5.5 kDa mass reduction observed for TGFR-3-ZP(ΔC) in the mass spectrometry analysis results from a C-terminal truncation of TGFR-3-ZP. At two positions, the amino acids could not be identified unambiguously by Edman sequencing (X = any amino acid). (C) Digestion of recombinant His-tagged TGFR-3-ZP protein with limiting concentrations of the unspecific serine protease subtilisin instead of proteinase K. Samples were retrieved at indicated time intervals and analyzed with SDS-PAGE. As for proteinase K, subtilisin treatment of TGFR-3-ZP generates one major proteolysis resistant fragment (black triangle). This fragment is highly similar to the fragment obtained after digestion with proteinase K (Fig. 3). (TIF) [file pone.0067214.s002.tif]

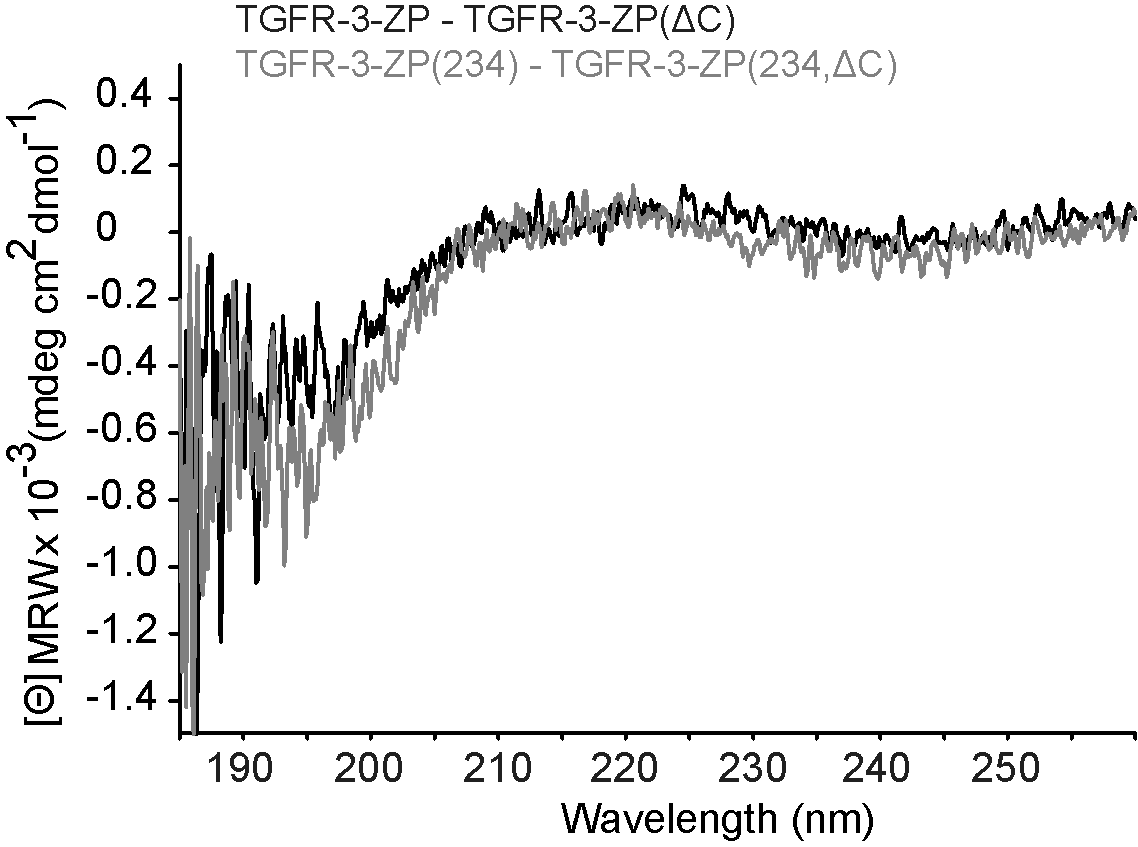

Supplement: Figure S3 — Circular dichroism difference spectra of TGFR-3 variants. CD difference spectra were obtained by subtracting the spectra of TGFR-3-ZP(ΔC) and TGFR-3-ZP(234,ΔC from the spectra of TGFR-3-ZP and TGFR-3-ZP(234), respectively. The difference spectra resemble the CD spectrum of a protein devoid of secondary structure elements. This possibly hints that the C-terminally cleaved-off fragments are largely disordered. (TIF) [file pone.0067214.s003.tif]

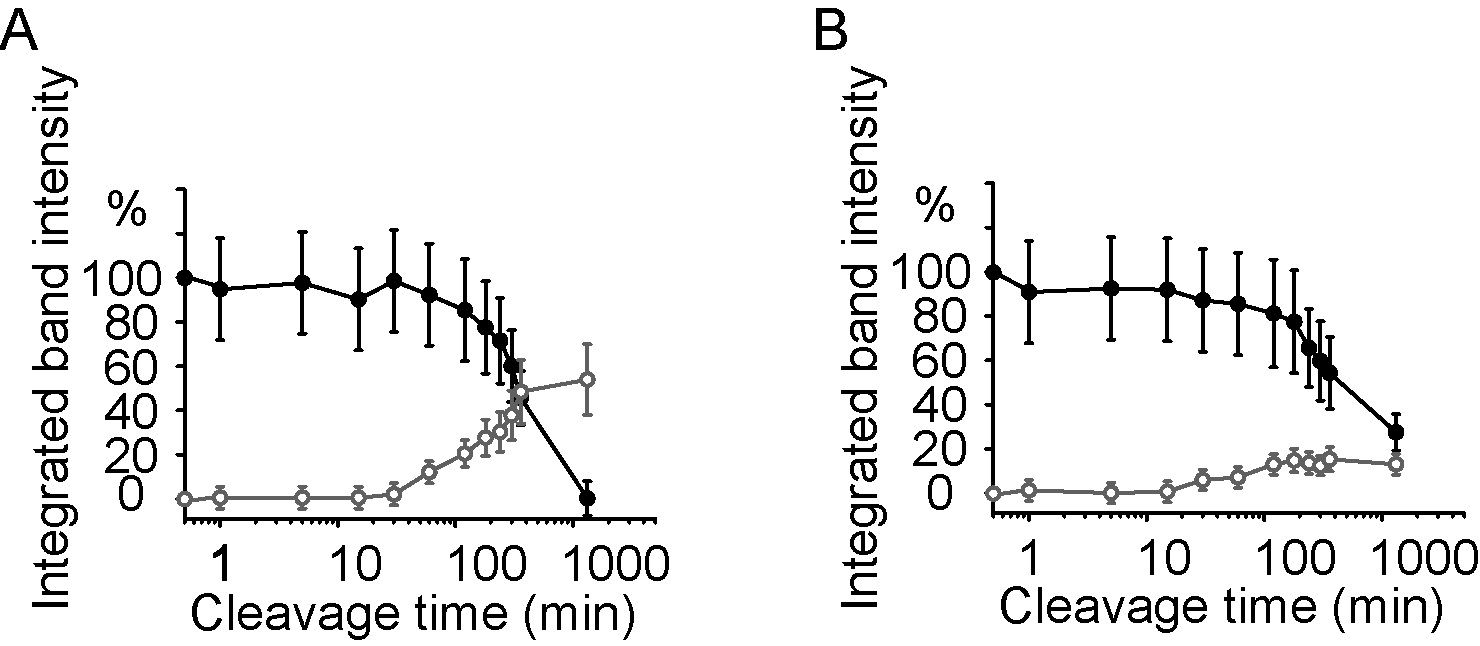

Supplement: Figure S4 — Video-densitometric quantification of protein bands in SDS gels. (A) Band intensities obtained in the SDS gels after limited proteolysis of TGFR-3-ZP with proteinase K (see Fig. 3A, main text) were analyzed by video-densitometry. (B) Same as for (A) but referring to the sample that was incubated with TGF-β2 prior to protease exposure (see Fig. 3C, main text). Full-length TGFR-3-ZP protein, black line; proteolytic fragment TGFR-3-ZP core, grey line. (TIF) [file pone.0067214.s004.tif]

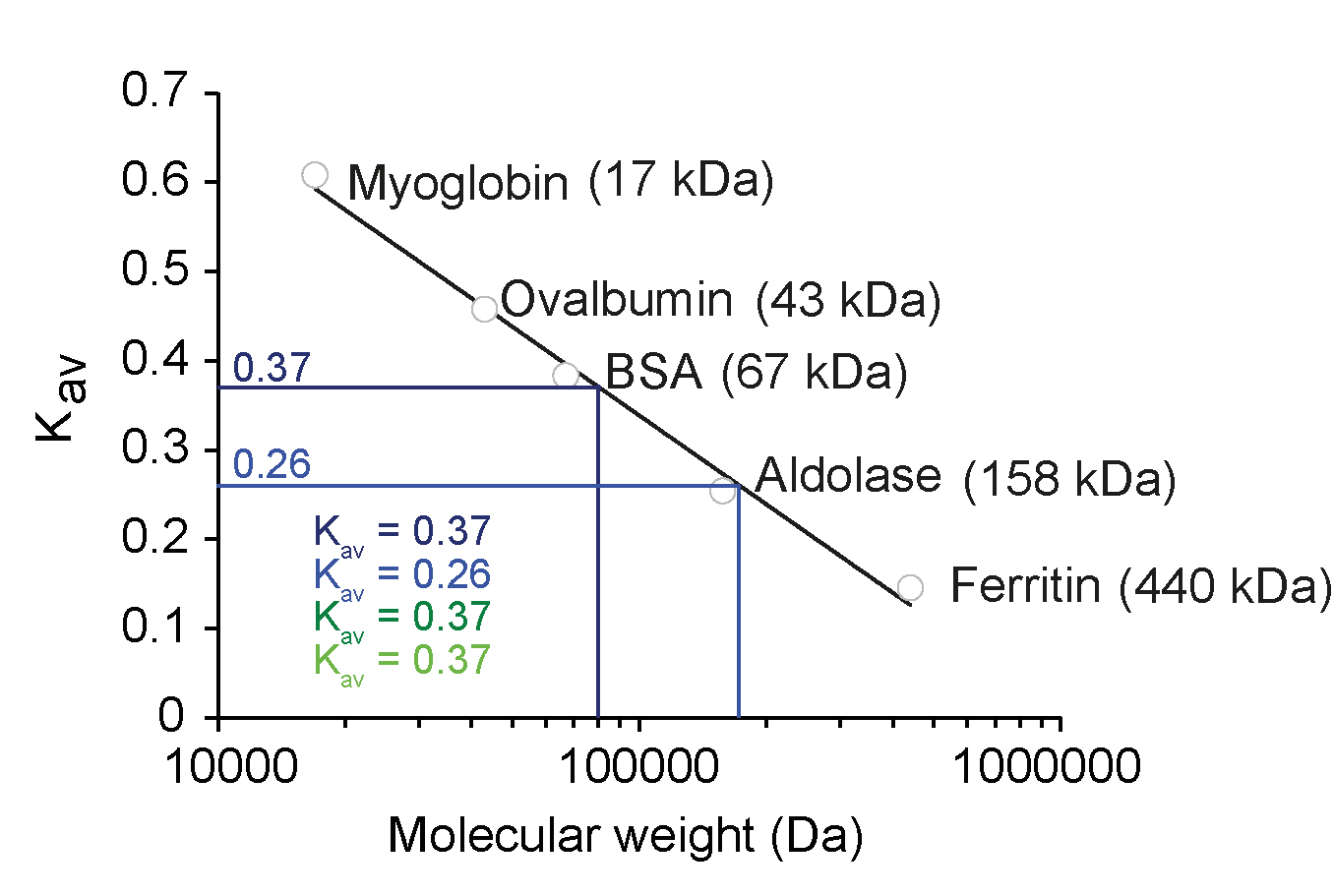

Supplement: Figure S5 — Molecular weight calibration curve used for the estimation of the oligomeric state of various protein samples. The calibration curve was derived from the elution profile of reference proteins (Fig. 4A, main text). The standard proteins’ partitition coefficients (Kav) are plotted against the specific molecular weights. The Kav-values determined for TGFR-3-ZP and TGFR-3-ZP(ΔC) are marked for the free forms and the samples that have been incubated with TGF-β2. Colours are as in Fig. 4A (main text). (TIF) [file pone.0067214.s005.tif]

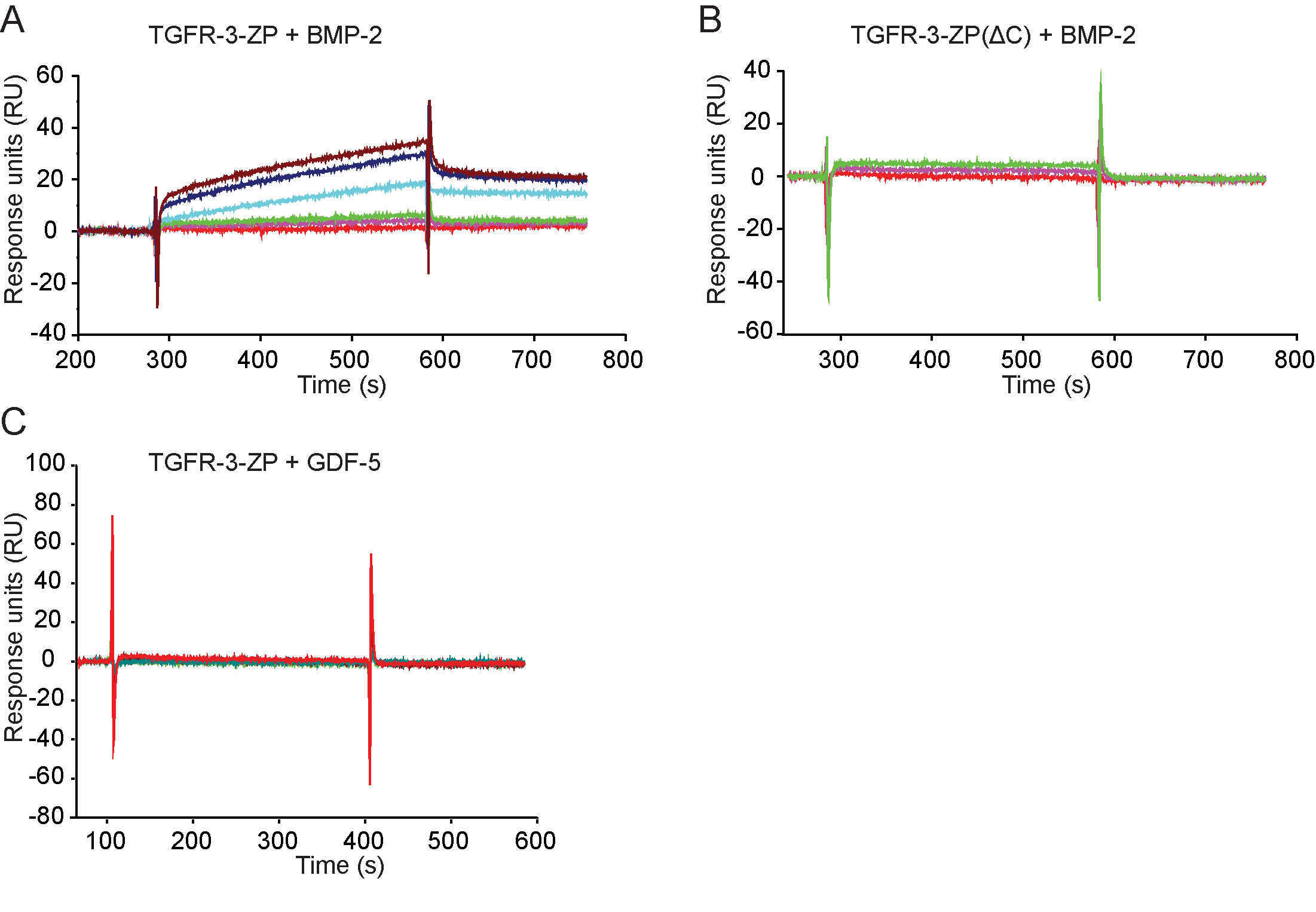

Supplement: Figure S6 — SPR measurements with TGFR-3-ZP and TGFR-3-ZP(ΔC). (A) TGFR-3-ZP and immobilized BMP-2, (B) TGFR-3-ZP(ΔC) and immobilized BMP-2 and (C) TGFR-3-ZP and immobilized GDF-5. (TIF) [file pone.0067214.s006.tif]

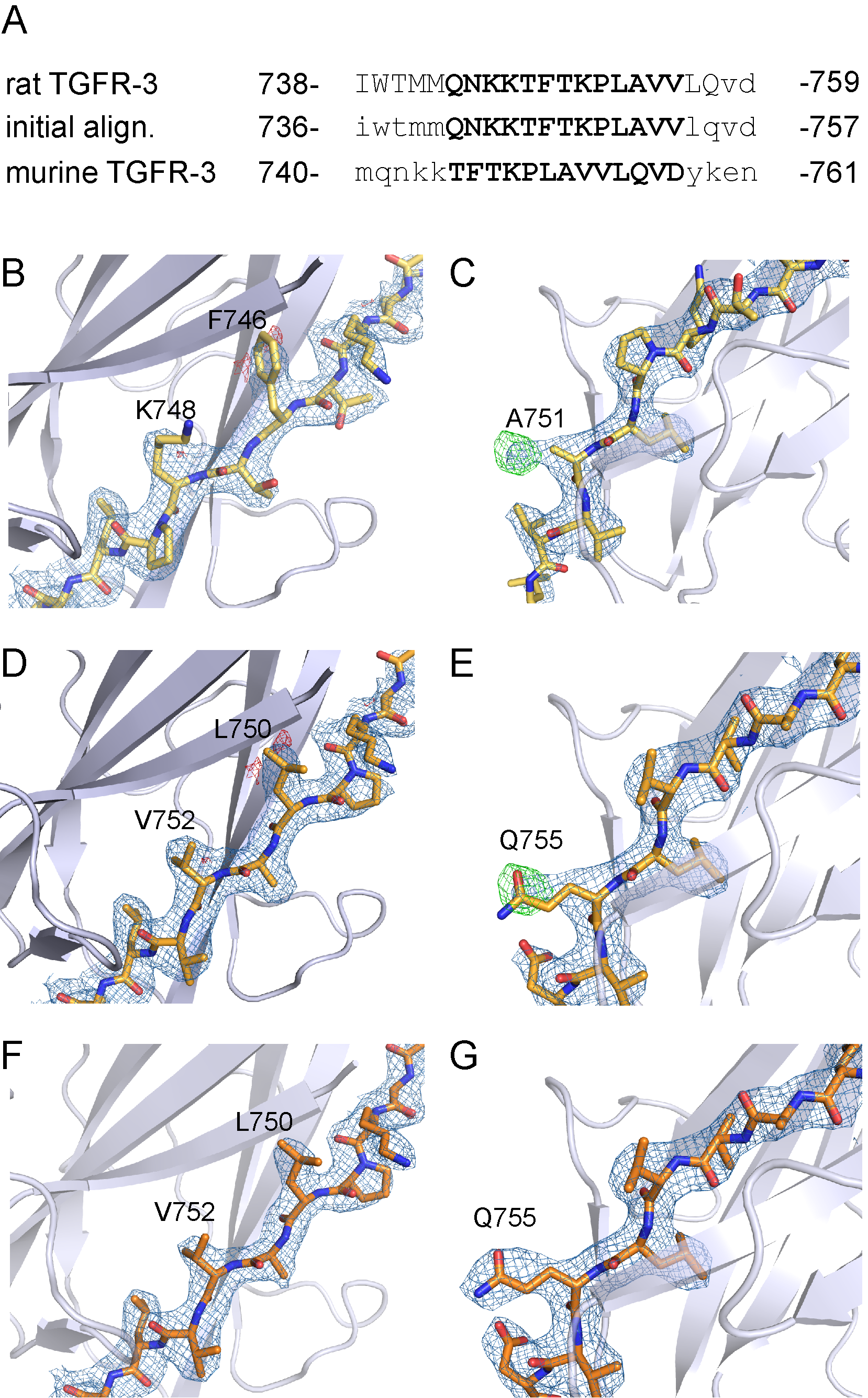

Supplement: Figure S7 — Shift of β-strand G registration and of the EHP sequence in the murine TGFR-3-ZP-C structure when compared to the structure of rat TGFR-3-ZP-C. (A) Structural alignment of the EHP of rat (upper line) and murine TGFR-3-ZP-C (lower line). Whereas the overall sequence identity between rat and murine TGFR-3-ZP (residues 590 to 755, murine numbering) is 97%, the identity is 100% in the displayed segment (middle line). Hence, the sequence assignment in this segment in the first murine ZP-C model was kept identical to that in the rat ZP-C structure (PDB entry entry 3QW9). However, in subsequent rounds of crystallographic refinement it became obvious that the sequence in the murine ZP-structure has to be offset by four residues when compared to the rat structure (see also panels B to F). Although the sequence identity of the structurally aligned sequences is now as low as 23%, the sequence similarity is still in the order of 40% (as reported by the SIAS server, http://imed.med.ucm.es/Tools/sias.html). In lower letters, amino acid residues not visible in the electron density maps of either the murine or rat ZP-C structure. In bold, residues forming the extended strand G in ZP-C, which includes the EHP sequence. (B and C) 2mFo-DFc and mFo-DFc electron density in strand G in the murine ZP-C structure when using an identical amino acid registration in murine and rat TGFR-3-ZP-C. This registration leads to negative difference density at the position of the side chain of Phe746 (panel B) and unexplained positive electron density near residue Ala751 (panel C). The 2mFo-DFc electron density (in blue) is displayed at a 1.0 σ level in all panels. The positive and negative difference electron density of the mFo-DFc electron density map in this and subsequent panels is displayed at 3.0 (in green) and -3.0 sigma levels (in red), respectively. (D and E) A four residue shift in the registration of the protein sequence explains the 2mFo-DFc and mFo-Fc electron density in this segment bette [file pone.0067214.s007.tif]
